# Comparisons of parallel potential biomarkers of _1_H-MRS-measured hepatic lipid content in patients with non-alcoholic fatty liver disease

**DOI:** 10.1038/srep24031

**Published:** 2016-04-15

**Authors:** Kai-Lun Shih, Wei-Wen Su, Chia-Chu Chang, Chew-Teng Kor, Chen-Te Chou, Ting-Yu Chen, Hung-Ming Wu

**Affiliations:** 1 Department of Gastroenterology, Changhua Christian Hospital, Changhua, Taiwan; 2 Department of Nephrology, Changhua Christian Hospital, Changhua, Taiwan; 3 School of Medicine, Chung-Shan Medical University, Taichung, Taiwan; 4 Internal Medicine Research Center, Changhua Christian Hospital, Changhua, Taiwan; 5 Department of Medical Imaging, Changhua Christian Hospital, Changhua, Taiwan; 6 Inflammation Research & Drug Development Center, Changhua Christian Hospital, Changhua, Taiwan; 7 Department of Neurology, Changhua Christian Hospital, Changhua, Taiwan; 8 Graduate Institute of Acupuncture Science, China Medical University, Taichung, Taiwan

## Abstract

Non-alcoholic fatty liver disease (NAFLD) is the main cause of chronic liver disease. This cross-sectional study aimed to evaluate whether parallel clinical features and serum markers are related to the severity of NAFLD. We enrolled 111 participants with different metabolic syndrome (MetS) scores (zero, n = 22; one, n = 19; two, n = 22; and ≥ three, n = 48) and used _1_H-MRS to measure liver fat content. Biochemical profiles and potential biomarkers of NAFLD were measured in fasting plasma. We found that _1_H-MRS-measured fat content was significantly associated with MetS score ≥1, endotoxin, and hs-CRP. Ordinal logistic regression analysis revealed that MetS score ≥2 and endotoxin were predictive of NAFLD (_1_H-MRS > 5%) and that endotoxin, hs-CRP, and malondialdehyde (MDA) were predictive of NAFLD with liver injury (_1_H-MRS > 9.67%). Endotoxin plus MetS score was shown to be the most accurate predictor of overall NAFLD (AUC = 0.854; (95% CI: 0.785–0.924), *P* < 0.001), and endotoxin plus hs-CRP and MDA was found to be predictive of NAFLD with liver injury (0.868; (0.801–0.936), *P* < 0.001). These results suggest that MetS score plus certain serum biomarkers with _1_H-MRS findings may hold promise for developing an effective model for monitoring the severity of NAFLD.

Non-alcoholic fatty liver disease (NAFLD) is a condition in which triglycerides accumulate in the hepatocytes of patients without excessive alcohol consumption. The disease mainly affects overweight and obese individuals but is also found in lean people with metabolic syndrome (MetS)^1^. The prevalence of NAFLD is very high in North and South America, Europe, the Middle East and the Asia-Pacific region^2^,^3^,^4^,^5^ with a community prevalence ranging from 15% to 45% depending on the methods used to assess liver steatosis^4^,^5^. The spectrum of clinical and pathological conditions in patients with NAFLD ranges from fatty liver alone to non-alcoholic steatohepatitis (NASH) with or without fibrosis to hepatic cancer^6^,^7^. Recent studies have also shown that NAFLD is associated with components of metabolic syndrome such as insulin resistance, abdominal obesity and a pro-inflammatory state, and with increased risk of developing type 2 diabetes mellitus^8^,^9^.

The natural history and pathogenesis of NAFLD are not clearly understood. In 1988, Day *et al.* established the two-hit hypothesis to explain the pathogenesis of NAFLD^10^. According to that well-known hypothesis, the accumulation of hepatic fat sensitizes the liver to injury, resulting in an inflammatory response due to increased oxidative stress^10^. In 2010, Tilg and Moschen proposed that many hits acting in parallel better explain the development of fatty liver^11^. Among those parallel hits include lipid peroxidation, inflammation, adipocytokines, and mitochondria dysfunction, which result in hepatocyte damage and hepatic fibrosis^11^. Currently, liver biopsy is the only method with which to definitively monitor the progression of fatty liver. Biopsy, however, is invasive, carries a risk of post-procedural infection, and is contraindicated in certain patient groups. An alternative to biopsy is the measurement of biomarkers in the circulation. A number of biomarkers have been studied as potential predictors of the development and progression of fatty liver^12^,^13^,^14^. For example, C-reactive protein (CRP), an acute-phase reactant produced by the liver, has been shown to be an independent predictor of NASH as well as of the severity of fibrosis in patients with NASH^15^. Endotoxin, the major constituent of the outer cell wall in Gram-negative bacteria, has been found to be a potent inducer of inflammation in NAFLD^16^,^17^. Malondialdehyde (MDA), a maker of oxidative stress, has been demonstrated to be associated with fatty liver stage^18^. Although many biomarkers have been demonstrated to be associated with the development of fatty liver, few studies have evaluated the interplay among high-sensitivity CRP (hs-CRP), endotoxin, MDA and components of metabolic syndrome on the severity of hepatic fat content or whether combined biomarkers are sensitive and specific markers of fatty liver progression.

Several non-invasive imaging methods such as ultrasonography, computed tomography, magnetic resonance imaging (MRI), and proton magnetic resonance spectroscopy (_1_H-MRS) provide a non-invasive means with which to accurately quantify intrahepatic lipid content^19^,^20^,^21^. Among those imaging methods, _1_H-MRS is able to detect small amounts of intrahepatic lipid accumulation^19^ and is more accurate than liver biopsy for measuring changes in hepatic steatosis^22^. In a recent study, we found that gamma-induced protein 10, a pro-inflammatory factor, endotoxin and increased levels of oxidative stress were markers of fatty liver progression^23^. Herein, we used _1_H-MRS to define the status of fatty liver and then determined whether endotoxin, inflammation, oxidative stress, and metabolic components were predictive of _1_H-MRS-measured fatty liver and whether those factors were associated with different grades of _1_H-MRS-measured fat content in patients with NAFLD. To test our hypothesis, we measured and analyzed the differences in metabolic parameters, inflammatory factors, endotoxin, and biologic markers of oxidative stress in participants who had no history of systemic diseases, viral hepatitis, alcoholism, or usage of medications for hyperlipidemia.

## Results

### Demographic, Clinical and Laboratory Data

In the present study, we enrolled 111 participants who visited the Health Management Center at the Changhua Christian Hospital for health management reasons over a period of twenty months. First, we examined differences in degree of _1_H-MRS-measured fat content between the MetS score subgroups by one-way ANOVA and found no significant differences in content among MetS score subgroups 3, 4 and 5 (*P* = 0.815). Therefore, participants with those scores were placed in the same subgroup, namely the MetS score subgroup ≥3 (n = 49) (Table 1). In addition, among participants with _1_H-MRS-measured fat content values > 5%, we found that a _1_H-MRS-measured hepatic fat content value of 9.67% was the optimal cutoff value indicative of NAFLD with liver injury. Therefore, the severity of _1_H-MRS-measured fatty liver was trichotomized into (a) _1_H-MRS values ≤5%, indicating normal liver (b) _1_H-MRS values > 5% but ≤9.67%, indicating simple NAFLD, and (c) _1_H-MRS values > 9.67%, indicating NAFLD with liver injury.

Table 1 shows the demographic, clinical, and laboratory data on patients in the four MetS score subgroups. There were no significant differences in age, gender, or smoking status among the four subgroups. The percentage of participants with normal liver was highest in the MetS 0 group (81%) and that of participants with NAFLD plus liver injury was highest in the MetS ≥3 group (65.3%). The values of all tested variables were significantly higher in the MetS score ≥3 group than in the other three MetS subgroups (Table 1).

### Correlations between _1_H-MRS-measured fat content, inflammation, oxidative stress, components of metabolic syndrome, and liver enzymes

**Figure 1:**
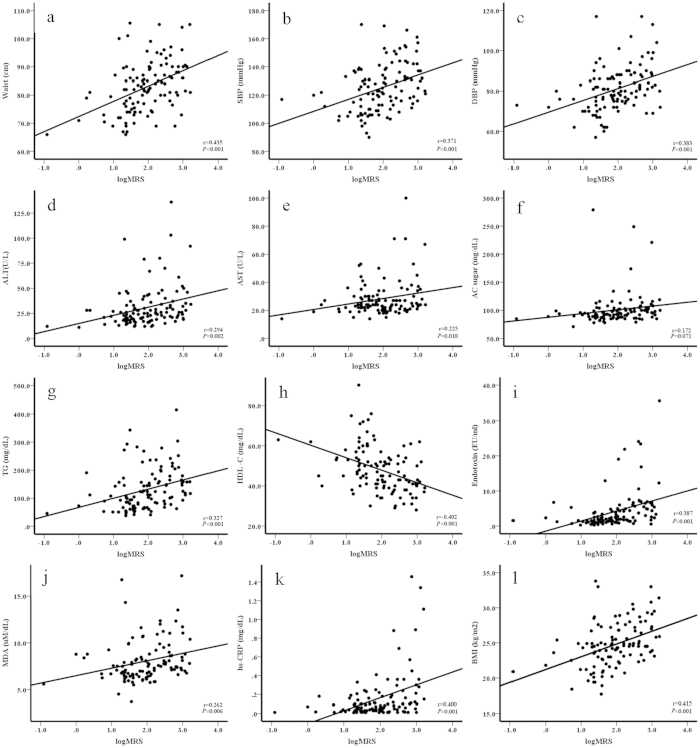
_1_H-MRS-measured fat content correlates with components of metabolic syndrome, oxidative stress, hs-CRP and endotoxin. Positive associations between _1_H-MRS-measured fat content and waist size (**a**), systolic blood pressure (SBP) (**b**), diastolic blood pressure (DBP) (**c**), alanine transaminase (ALT) (**d**), aspartate transaminase (AST) (**e**), fasting glucose (AC) (**f**), triglyceride (TG) (**g**), high-density cholesterol (HDL-C) (**h**), endotoxin (**i**), malondialdehyde (MDA) (**j**), high-sensitivity C-reactive protein (hs-CRP) (**k**), and body mass index (BMI) (**l**).

The results of the Pearson’s correlation test revealed that _1_H-MRS-measured hepatic fat content was positively correlated with circulating endotoxin (r = 0.387, *P* < 0.001), MDA (r = 0.262, *P* = 0.006), hs-CRP (r = 0.40, *P* < 0.001), body mass index (BMI) (r = 0.415, *P* < 0.001), waist circumference (r = 0.435, *P* < 0.001), aspartate transaminase (AST) (r = 0.225, *P* = 0.018), alanine transaminase (ALT) (r = 0.294, *P* = 0.002), systolic blood pressure (SBP) (r = 0.371, *P* < 0.001), diastolic blood pressure (DBP) (r = 0.383, *P* < 0.001), fasting glucose (r = 0.172, *P* = 0.071), triglyceride (TG) (r = 0.327, *P* < 0.001), and inverse high-density cholesterol (HDL-C) (r = 0.402, *P* < 0.001) (Fig. 1).

### Association between _1_H-MRS-measured fat content and circulating hs-CRP, endotoxin, and metabolic components

Significant variables in the univariate analyses were then included in a multivariate linear regression model to examine the most important predictors of _1_H-MRS-measured hepatic fat content. The analysis revealed that hs-CRP (*P* = 0.010), endotoxin (*P* = 0.026), and MetS scores 1 (*P* = 0.012), 2 (*P* = 0.001), and ≥3 (*P* = 9.55 × 10^−9^) were significantly associated with the grade of _1_H-MRS-measured fat content after adjusting for age, BMI, gender, white blood cell (WBC) count, and lipid profiles (Table 2).

### Multivariate ordinal logistic regression analysis to evaluate the associations among hs-CRP, endotoxin, and metabolic syndrome traits and degree of _1_H-MRS-measured hepatic fat content

Univariate logistic regression analysis revealed that MetS scores 2 and ≥3, endotoxin, hs-CRP, and WBC count were positively associated with the _1_H-MRS-measured degree of fatty liver. After adjusting for traditional risk factors such as age, sex, and smoking status and non-traditional possible confounders such as components of metabolic syndrome, WBC count, endotoxin, hs-CRP, and MDA, a MetS score of 2 (Odds Ratio (OR), 7.23; 95% confidence interval (CI), 1.70–30.78), MetS scores ≥3 (OR, 17.07; 95% CI, 3.81–76.46), and endotoxin (OR, 1.44; 95%CI, 1.05–1.98) remained significantly associated with the severity of _1_H-MRS-measured fatty liver (Table 3). Power analysis revealed that MetS score had a power of 99.8% and endotoxin had a power of 97.8% in differentiating between patients with _1_H-MRS values > 5% and those with the value ≤ 5%. Ordinal logistic regression analysis revealed that endotoxin (OR, 1.18; 95% CI, 1.06–1.32), hs-CRP (OR, 662.2; 95% CI, 11.68–37530.09) and MDA (OR, 1.39; 95% CI, 1.09–1.77) were predictive of _1_H-MRS-measured steatohepatitis (Table 3). Power analysis revealed that endotoxin had a power of 96.7%, hs-CRP had a power of 98.3% and MDA had a power of 95.9% in differentiating between patients with _1_H-MRS value > 9.67% and those with the value ≤ 9.67%.

### Circulating endotoxin combined with hs-CRP, MDA and MetS score is predictive of severity of _1_H-MRS-measured fatty liver

**Figure 2:**
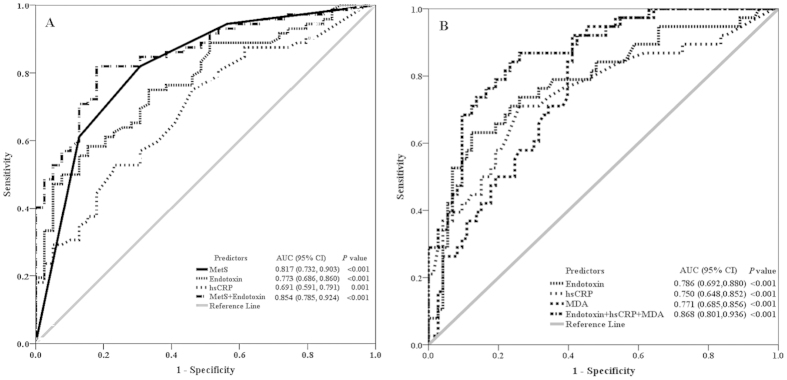
Accuracy of potential biomarkers in detecting the degree of _1_H-MRS-measured hepatic fat content. Receiver-operating characteristic curves for MetS score, MDA, endotoxin, and hs-CRP, and their combination in diagnosing overall non-alcoholic fatty liver (_1_H-MRS > 5%) (**A**) and non-alcoholic fatty liver with liver injury (_1_H-MRS > 9.67%) (**B**).

Receiver-operating characteristic (ROC) curves were constructed to evaluate whether endotoxin, hs-CRP, MDA, a biomarker of oxidative stress, and metabolic syndrome traits could serve as diagnostic biomarkers for the severity of _1_H-MRS-measured fatty liver. Endotoxin was a significant predictor of both overall _1_H-MRS-measured NAFLD (Fig. 2A) and NAFLD with liver injury (Fig. 2B), with areas under the ROC curve (AUC) of 0.773 (95% CI, 0.686–0.860; *P* < 0.001) and 0.786 (95% CI, 0.692–0.880; *P* < 0.001), respectively. Endotoxin combined with MetS score was an even more accurate diagnostic measure of fatty liver severity, with an AUC of 0.854 (95% CI, 0.785–0.924; *P* < 0.001) (Fig. 2A). The measure with the highest accuracy for diagnosing NAFLD with liver injury was endotoxin combined with hs-CRP and MDA (AUC, 0.868; 95% CI, 0.801–0.936; *P* < 0.001) (Fig. 2B).

## Discussion

In the present study, we evaluated the interplay among biomarkers associated with _1_H-MRS-measured hepatic fat content and whether combined biomarkers are sensitive and specific diagnostic markers of fatty liver progression in participants without alcohol abuse. We found that participants with higher metabolic scores had significantly higher levels of _1_H-MRS-measured fat content (*P* = 2.19 × 10^−10^ and *P*-trend = 4.69 × 10^−13^). In addition, multiple regression analysis showed that MetS scores ≥1, endotoxin, and hs-CRP were significantly associated with the degree of _1_H-MRS-measured fat content. Furthermore, ordinal logistic regression analysis revealed that MetS scores 2 and ≥3 and endotoxin were independent predictors of overall NAFLD (_1_H-MRS > 5%) and that MDA, endotoxin, and hs-CRP were predictive of NAFLD with liver injury (_1_H-MRS > 9.67%). ROC analysis demonstrated that endotoxin combined with hs-CRP and MDA (*P* < 0.001) was the most accurate measure for diagnosing fatty liver with hepatic dysfunction (Fig. 2).

Studies have shown that multiple parallel hits contribute to hepatic steatosis and its progression to hepatic fibrosis^11^,^24^. In the present study, we used _1_H-MRS to measure the degree of hepatic fat content and found that MetS score, endotoxin, hs-CRP, and MDA are closely associated with the severity of fatty liver. Endotoxin, hs-CRP, and MDA were found to be associated with the progression of NAFLD in our previous study that measured fatty liver content on MR images^23^ as well as in several large studies that measured fat content in liver biopsy specimens^25^,^26^,^27^. A number of studies have provided evidence that gut-derived factors, systemic inflammation, and oxidative stress play important roles in the pathogenesis of NAFLD^24^,^12^. In addition, studies have shown that metabolic syndrome in general and its components specifically are risk factors for NAFLD^28^,^29^. In the present study, each component of metabolic syndrome was closely correlated to values of _1_H-MRS-measured fat content (Table 1 and Fig. 1). We found that metabolic syndrome was a consistent risk factor for fatty liver and that a MetS score ≥1 is a potential predictor of fatty liver (Tables 2 and 3). The three parallel biomarkers endotoxin, hs-CRP, and MDA, as well as a MetS score ≥1 were all predictive of the degree of fatty liver and therefore might provide an alternative measure for staging the severity of NAFLD.

Imaging modalities such as ultrasonography, computed tomography, MRI and MRS are increasingly used as alternatives to biopsy, particularly for assessing the presence and severity of steatosis and nonalcoholic steatohepatitis^30^. Of those imaging methods, MRS is the most sensitive in detecting low-grade fat infiltration in liver and shows a good correlation with the results of liver biopsy^19^,^31^,^32^. In the present study, _1_H-MRS-measured hepatic fat content was highly correlated with levels of inflammatory markers (endotoxin and hs-CRP), metabolic components, oxidative stress (MDA), and liver injury (ALT and AST) (Fig. 1). Our results also support that MRS is an accurate method for detecting fatty liver alone as well as fatty liver with hepatic dysfunction, indicating that the technique can be used to both detect and monitor NAFLD and its progression. MRS provided us with the opportunity to identify the contribution of potential biomarkers of NAFLD severity. We found that endotoxin, hs-CRP, and MDA, but not MetS score, were highly associated with _1_H-MRS-diagnosed steatohepatitis (_1_H-MRS > 9.67%). However, the accuracy of _1_H-MRS for detecting the degree of hepatic fat content needs to be validated by comparison with biopsy specimens before the modality can be used in routine clinical practice.

Liver biopsy is the gold standard for diagnosing NAFLD and its progression to steatohepatitis and fibrosis; however, the procedure is not indicated in all patients because of its invasive nature and the potential risks associated with the procedure^33^,^34^. A number of non-invasive measures have been developed to assess the presence and severity of NAFLD including tests of serum biomarkers (e.g. cytokines) and imaging modalities (e.g. MRI and MRS)^13^,^14^. Recent studies have provided evidence that a combination of clinical features, serum biomarkers and imaging techniques can improve the accuracy of differentiating steatosis from steatohepatitis^35^,^36^,^37^. Several diagnostic panels have been shown to be able to discriminate between simple NAFLD (steatosis) and NASH, namely the HAIR score, which includes 3 variables (hypertension, ALT insulin resistance)^38^, the NashTest^®^, which measures 13 variables (weight, triglycerides, glucose, α2-macroglobulin and apolipoprotein)^39^, the SteatoTest^®^, which comprises 10 variables (simple blood tests, age, gender and BMI)^40^, and the hepatic steatosis index (HSI), which measures 4 variables (ALT/AST, BMI, gender, and diabetes)^41^. Although studies have shown that those panels are reasonably accurate predictors of either steatosis or NASH, none of the tests have been shown to accurately quantify steotosis, thereby limiting their clinical utility^35^,^36^,^37^. A growing understanding of the pathophysiology of NAFLD has allowed researchers to study more specific, mechanism-based biomarkers that could provide a reliable noninvasive alternative to liver biopsy. The present and previous studies have shown that there is a significant correlation between the presence of endotoxin, hs-CRP, or MDA in the circulation and histological findings characteristic of NAFLD or NASH^25^,^26^,^27^ However, few studies have provided evidence that simultaneous measurement of these parallel biomarkers improves the accuracy of diagnosing NAFLD. To the best of our knowledge, this study is the first to report that the presence of circulating endotoxin combined with MetS score (AUC, 0.854) is significantly predictive of overall NAFLD (Fig. 2A) and that MetS score combined with endotoxin, hs-CRP and MDA (AUC, 0.865) is highly predictive of NAFLF with liver injury (Fig. 2B). Our findings show that a diagnostic model comprised of MRS, MetS score and those three serum biomarkers may provide an effective strategy for detecting and monitoring the development and progression of fatty liver.

We also compared the diagnostic performance of the biomarkers identified in the present study with that of previously published biomarkers or scores for predicting the severity of _1_H-MRS-measured fatty liver. The HSI score^41^, the triglyceride x glucose (TyG) index^42^, and the visceral adiposity index (VAI), which measures 4 variables (waist circumference, triglyceride, HDL-C, and BMI)^43^ have been reported to have good diagnostic performance for predicting fatty liver^35^. We found that the AUCs for predicting overall _1_HMRS-diagnosed NAFLD and NAFLD with liver injury were 0.726 (95%CI, 0.612–0.832) and 0.800 (95%CI, 0.718–0.881) for HSI, 0.746 (95%CI, 0.643–0.850) and 0.776 (95%CI, 0.691–0.861) for the TyG index, and 0.768 (95%CI, 0.672–0.864) and 0.801 (95%CI, 0.717–0.884) for VAL (Supplementary Figure 1). In comparison, our results indicate that our model comprised of endotoxin with MetS score (AUC, 0.854) has a higher sensitivity for predicting NAFLD (Fig. 2A) and that our model comprised of MetS score combined with endotoxin, hs-CRP and MDA has a higher specificity for predicting NAFLD with liver injury (AUC, 0.865) (Fig. 2B) than all of the above-mentioned panels (Supplementary Figure 1).

There are several limitations in this study. First, this study was a cross-sectional study, and therefore does not allow for the inference of causal relationships. Second, the sample size in the study was relatively small, which may have reduced the statistical power. Further longitudinal studies with a larger sample size are needed to establish cause–effect relationships. Third, the detection of lipid content was based on MRS findings instead of liver biopsy. Although MRS findings are known to correlate with histopathologic characteristics of steatosis, MRS imaging can underestimate the condition in patients with moderate or advanced steatosis, such as necro-inflammation and fibrosis^44^. Fourth, alcohol intake, a common cause of fatty liver, was self-reported. Therefore, it is possible that not all patients in this study had NAFLD. Nonetheless, serum levels of gamma glutamyl transferase, a well-known biomarker of excessive alcohol consumption, were less than 100 U/L in all of the participants.

In summary, we found that MetS score, endotoxin, oxidative stress, and hs-CRP were closely associated with _1_H-MRS-measured hepatic fat content. Specifically, we found that MetS scores ≥2 combined with endotoxin were predictive of the presence of fatty liver (_1_H-MRS > 5%) and that the combination of endotoxin with hs-CRP and MDA was the most accurate measure for diagnosing NADLD with liver injury (_1_H-MRS > 9.67%). A diagnostic model comprised of MRS, MetS score and serum biomarkers may provide an effective strategy for detecting and monitoring the development and progression of fatty liver. Longitudinal studies are needed to support our novel findings.

## Participants and Methods

### Study design and participants

This cross-sectional case-control study was conducted at the Health Examination Center of the Changhua Christian Hospital during the period February 1, 2014 to September 30, 2015. All participants aged 45 to 60 years were eligible for inclusion. Exclusion criteria included evidence of systemic diseases, alcoholism, viral hepatitis type B or C infection, or HIV infection. Patients were also excluded if they were receiving medications for diabetes or hyperlipidemia, antioxidants (e.g. vitamins C and E), immunosuppressants, non-steroidal anti-inflammatory agents or hepatotoxic drugs. Waist circumference and blood pressure were measured in all subjects and all of the participants underwent _1_H-MRS to measure levels of hepatic lipid content. Blood specimens were also obtained from all participants after an overnight fast for anthropometric measurements and to measure biochemical and metabolic syndrome-associated profiles as well as endotoxin, hs-CRP, and MDA levels. The study was carried out in strict accordance with guidelines for research involving human subjects developed by the Taiwan Ministry of Health and Welfare. All experimental protocols were approved by the Institutional Review Board of the Changhua Christian Hospital (approval number 110507) and all of the participants provided written informed consent to participate in the study.

### Definition of metabolic syndrome (MetS) scores

Modified National Cholesterol Education Program (NCEP) criteria with Asian-specific cut-offs were used to evaluate whether patients had metabolic syndrome^45^. The syndrome was diagnosed in patients with three or more of the following MetS components: waist circumference (≥90 cm for men and ≥80 cm for women), blood pressure (systolic ≥130 mm Hg and/or diastolic ≥85 mm Hg), fasting glucose (≥100 mg/dl), high density cholesterol (<40 mg/dl in men and <50 mg/dl in women), or triglyceride (≥150 mg/dl)^24^. Patients were initially stratified into MetS score subgroups according to the number of MetS components.

### Proton magnetic resonance spectroscopy (_1_H-MRS) to determine liver fat content

Single breath-hold single-voxel _1_H-MRS data were acquired in mid-expiration using point-resolved single voxel spectroscopy pulse sequence with the following parameters: TR, 1500 ms; TE, 30 ms; total acquisition time, 18s. On the spatially localized three-dimensional T2-weighted images of liver, a 30 mm × 30 mm × 30 mm single block was positioned on the right anterior lobe, with care taken to avoid large luminal structures. Syngo software (Syngo Spectroscopy Evaluation, Siemens Medical Systems, Erlangen, Germany) was used to measure the peak height of the water peak at 4.7 ppm and the lipid peak at 1.2 ppm. Intrahepatic lipid content on _1_H-MRS images was calculated as follows: Fat content = lipid/(lipid + water) × 100%^19^,^20^. MRS results were analyzed by a radiologist with 9 years of experience who was blinded to all clinical and laboratory findings. In the present study, MRS-measured fat content ≤5% was defined as normal liver.

### Carotid ultrasonography to measure intima-media thickness of the common carotid arteries

Intima-media thickness was assessed using high resolution B-mode ultrasonography (Acuson 128XP, equipped with a 7-MHz linear-array transducer). The intima-media thickness (IMT) value was defined as the mean IMT of the right and left common carotid arteries and was calculated from 10 measurements on each side taken 10 mm proximal to the carotid bifurcation. Lumen/intima leading edge (I-line) to media/adventitia leading edge (M-line) was validated as previously described^46^. All ultrasonographic measurements were performed by the same experienced sonographer.

### Assays for circulating endotoxin

Plasma endotoxin levels were measured by a chromogenic Limulus Amebocyte Lysate assay (QCL-1000™; Lonza, Walkersville, MD, USA). All assays were performed in accordance with the manufacturer’s instructions.

### Assays for plasma malondialdehyde

Malondialdehyde (MDA) is one of the most frequently used indicators of oxidative stress^47^. Plasma MDA was assayed with a thiobarbituric acid reactive substances assay kit (Cayman Chemical Company, Ann Arbor, MI, USA) according to the manufacturer’s instructions. Absorbance of the samples was measured at 532 nm by a microplate reader (Versa Max, Molecular Devices, Sunnyvale, CA, USA). The concentration of MDA was determined using an MDA standard curve.

### Other measures

Serum aspartate transaminase (AST), alanine transaminase (ALT), fasting glucose, glycated hemoglobin (HbA1c), high sensitivity C-reactive protein (hs-CRP) and lipid profiles including total cholesterol, triglyceride, low-density lipoprotein cholesterol (LDL-C) and high-density lipoprotein cholesterol (HDL-C) were measured using standard procedures at the Department of Laboratory Medicine, Changhua Christian Hospital.

### Statistical analysis

Data are presented as a percentage, median (interquartile range) or mean ± standard deviation. Each variable was tested for normal distribution using the Kolmogorov-Smirnov test. We used the chi-square test for categorical comparisons of data and the ANOVA test or the Kruskal–Wallis test to measure differences in means of continuous variables between the four MetS score subgroups. The Pearson correlation test was performed to test the correlation between _1_H-MRS-measured fat content and clinical and metabolic profiles (Fig. 1). Significant variables in the univariate analyses were then included in a multiple linear regression model to assess the relationships between _1_H-MRS-measured fat content, MetS score, and potential serum biomarkers (Table 2). Youden’s index^48^ was used to determine the optimal cut-off value of _1_H-MRS for fatty liver with liver injury (ALT level ≥40 U/L). Significant variables in the univariate analyses were also included in a multivariate ordinal logistic regression model to assess the association between MetS score, potential biomarkers and two subgroups of _1_H-MRS-measured fat content, namely _1_H-MRS > 5%, indicating overall NAFLD and _1_H-MRS > 9.67%, indicating NAFLD with liver injury (Table 3). _1_H-MRS-measured values were log-transformed (In) in the correlation test and in the linear regression model. A *P*-value of <0.05 was considered to indicate statistical significance. All statistical analyses were performed using the statistical package SPSS (IBM SPSS Statistics, version 20, IBM Corporation, Chicago, IL, USA).

## Additional Information

**How to cite this article**: Shih, K.-L. *et al.* Comparisons of parallel potential biomarkers of _1_H-MRS-measured hepatic lipid content in patients with non-alcoholic fatty liver disease. *Sci. Rep.* **6**, 24031; doi: 10.1038/srep24031 (2016).

## Supplementary Material

Supplementary Information

## Figures and Tables

**Table 1:** Demographic, clinical, and laboratory data of the four subgroups of participants stratified by metabolic syndrome score.

	MetS 0	MetS 1	MetS 2	MetS ≥3	*P*-value	*P*_-trend_
n	21	19	22	49	–	–
Male, n (%)	7 (33.33%)	10 (52.63%)	15 (68.18%)	30 (61.22%)	0.097	ND
Age, years	49 (46,54)	50 (46,55)	53.5 (46,58)	52 (47,56)	0.294	ND
BMI, kg/m^2^	21.8 (20.2,23.5)	24.2 (22.1,24.9)	24.35 (22.5,25.8)	25.9 (24.5,28.7)	1.05 × 10^−6^	1.40 × 10^−8^
SBP, mm Hg	107.52 ± 8.9	122.74 ± 13.08	123.09 ± 13.18	134.61 ± 15.94	4.39 × 10^−10^	2.42 × 10^−10^
DBP, mm Hg	71.14 ± 6.36	78.26 ± 7.85	79.32 ± 8.66	87.04 ± 11.56	5.33 × 10^−8^	2.61 × 10^−8^
Waist, cm	74.6 ± 6.28	78.66 ± 6.02	83.89 ± 7.73	87.69 ± 8.55	4.73 × 10^−9^	3.17 × 10^−10^
Smoking	1 (4.76%)	1 (5.26%)	2 (9.09%)	8 (16.33%)	0.389	ND
WBC, mm^3^	4.8 (4,5.1)	4.5 (3.8,5.7)	5.15 (4.5,6)	5.7 (5.2,7)	7.44 × 10^−5^	6.28 × 10^−6^
AST, U/L	24 (20,29)	23 (22,27)	25.5 (23,28)	24 (21,36)	0.291	0.095
ALT, U/L	20 (14,25)	24 (17,29)	24.5 (19,36)	29 (22,46)	1.24 × 10^−3^	5.94 × 10^−5^
Uric acid, mg/dl	5.1 ± 1.4	5.5 ± 1.4	6.25 ± 1.44	6.41 ± 1.28	1.19 × 10^−3^	8.09 × 10^−5^
Creatinine, mg/dl	0.7 (0.66,0.75)	0.76 (0.61,0.92)	0.84 (0.68,0.91)	0.8 (0.61,0.92)	0.371	0.168
AC, mg/dl	89 (85,92)	93 (90,97)	92 (87,99)	102 (95,114)	2.75 × 10^−7^	8.14 × 10^−9^
Hemoglobin A1c, %	5.2 (5,5.3)	5.5 (5.1,5.6)	5.45 (5.3,5.7)	5.7 (5.3,6)	1.94 × 10^−5^	8.37 × 10^−7^
Cholesterol, mg/dL	190.48 ± 31.01	212.74 ± 32.37	197.91 ± 46.43	210 ± 38.2	0.147	0.173
Triglyceride, mg/dL	65 (53,90)	81 (64,124)	104 (79,154)	159 (118,213)	9.43 × 10^−10^	9.87 × 10^−12^
HDL-C, mg/dL	57 (52,62)	51 (44,65)	45 (40,53)	41 (37,50)	7.33 × 10^−7^	3.10 × 10^−8^
LDL-C, mg/dL	114.99 ± 26.63	134.63 ± 27.91	124.95 ± 35.66	131.44 ± 38.6	0.231	0.174
Endotoxin, EU/ml	1.22 (0.9,1.6)	2.11 (1.27,2.66)	1.91 (1.09,2.98)	4.4 (1.9,6.93)	2.51 × 10^−6^	6.79 × 10^−8^
hs-CRP, mg/dl	0.03 (0.01,0.06)	0.03 (0.01,0.08)	0.07 (0.03,0.16)	0.12 (0.06,0.33)	8.51 × 10^−7^	1.50 × 10^−8^
MDA, μm/ml	6.69 (6.26,7.71)	6.74 (6.17,8.08)	7.07 (6.56,8.24)	8.09 (7.33,10.71)	1.55 × 10^−3^	6.84 × 10^−5^
Right IMT, mm	0.63 ± 0.08	0.64 ± 0.08	0.7 ± 0.12	0.74 ± 0.14	7.99 × 10^−4^	1.38 × 10^−4^
Left IMT, mm	0.62 ± 0.11	0.64 ± 0.08	0.72 ± 0.13	0.78 ± 0.14	2.65 × 10^−6^	3.80 × 10^−7^
_1_H-MRS, %	3.9 (2.66,4.8)	4.9 (3.87,8.04)	6.31 (4.91,7.64)	12.38 (7.8,17.6)	2.19 × 10^−10^	4.69 × 10^−13^
_1_H-MRS subgroups					1.14 × 10^−10^	1.01 × 10^−11^
_1_H-MRS ≤5	17 (80.95%)	10 (52.63%)	7 (31.82%)	5 (10.20%)	–	–
_1_H-MRS > 5 but ≤9.67	4 (19.05%)	7 (36.84%)	12 (54.55%)	11 (22.45%)	–	–
_1_H-MRS > 9.67	0 (0%)	2 (10.53%)	3 (13.64%)	33 (67.35%)	–	–

Data are presented as mean ± SD, median (Q1, Q3), or n (%) for categorical data. Differences in mean/median values of variables between the four MetS-stratified subgroups were tested by one-way ANOVA or the Kruskal-Wallis test. *P* for trend was calculated by the Jonckheere-Terpstra Test to test for an ordered alternative hypothesis within four groups.

Abbreviations: MetS, metabolic syndrome score; Q1, 25^th^ percentile; Q3, 75^th^ percentile; ND, not done; BMI, body mass index; SBP, systolic blood pressure; DBP, Diastolic blood pressure; WBC, white blood cell; ALT, alanine aminotransferase; AST, aspartate aminotransferase; AC, fasting sugar; HDL-C, high density lipoprotein cholesterol; LDL-C, low density lipoprotein cholesterol; hs-CRP, high-sensitivity C-reactive protein; MDA, malondialdehyde; IMT, intima media thickness of the common carotid artery; _1_H-MRS, proton magnetic resonance spectroscopy.

**Table 2:** Association between hs-CRP, endotoxin and _1_H-MRS-measured fat content in the entire study.

Variables	Entire study (n = 111)	
	Standardized RegressionCoefficients	*P* value
MetS score		
0	reference value	
1	0.227	0.012
2	0.323	0.001
≥3	0.676	9.55 × 10^−9^
Endotoxin	0.175	0.026
hs-CRP	0.202	0.010

_1_H-MRS values were log-transformed in the linear regression model.

Multivariate linear regression model with backward elimination selection was carried out.

Covariates entered into the model included MetS score, endotoxin, hs-CRP, MDA, age, gender, HbA1c, Tchol, LDL, BMI, and WBC.

Abbreviations: MetS score, metabolic syndrome score; hs-CRP, high-sensitivity C-reactive protein, MDA, malondialdehyde; HbA1c, Hemoglobin A1c; Tchol, total cholesterol; LDL, low density lipoprotein cholesterol; BMI, body mass index; and WBC, white blood cell.

**Table 3:** Ordinal logistic regression analysis of risk factors associated with _1_H-MRS-measured fatty liver.

Variables	_1_H-MRS > 5% vs MRS ≤ 5%				_1_H-MRS > 9.67% vs _1_H-MRS ≤ 9.67%			
	unadjusted OR (95% CI)	*p*-value	adjusted OR (95% CI)^a^	*p*-value	unadjusted OR (95% CI)	p-value	adjusted OR (95% CI)^b^	*p*-value
MetS								
1 vs 0	3.83 (0.93,15.72)	0.063	2.92 (0.68,12.60)	0.150		*inestimable*†		
2 vs 0	9.11 (2.22,37.34)	0.002	7.23 (1.70,30.78)	0.007				
≥3 vs 0	37.4 (8.96,156.13)	<0.001	17.07 (3.81,76.46)	<0.001				
Endotoxin	1.72 (1.28,2.31)	<0.001	1.44 (1.05,1.98)	0.024	1.22 (1.07,1.38)	0.003	1.18 (1.06,1.32)	0.004
hs-CRP	526.6 (4.21,65861.86)	0.011			905.82 (19.41,42271.81)	0.0005	662.2 (11.68,37530.09)	0.002
MDA	1.21 (0.98,1.5)	0.076			1.46 (1.17,1.82)	0.0007	1.39 (1.09,1.77)	0.008
Age	1.01 (0.94,1.09)	0.832			1 (0.93,1.08)	0.922		
Gender	1.56 (0.71,3.42)	0.266			1.34 (0.6,2.96)	0.475		
WBC	1.44 (1.06,1.94)	0.019			1.54 (1.16,2.04)	0.003		
Smoking	1.71 (0.44,6.74)	0.440			1.43 (0.42,4.85)	0.567		

Multivariate logisitc regression model with backward elimination selection was carried out.

^*a*^Covariates in the full model included MetS, endotoxin, hs-CRP, MDA, WBC, age, sex, and smoking.

^*b*^Covariates included endotoxin, hs-CRP, MDA, WBC, age, sex, and smoking.

^†^Odds ratios could not be calculated because none of the participants with MetS 0 had high-grade fatty liver (_1_H-MRS > 9.67%).
