# Supplementary material for: Comparisons of parallel potential biomarkers of 1H-MRS-measured hepatic lipid content in patients with non-alcoholic fatty liver disease
Source: Sci Rep. 2016 Apr 15;6:24031. doi: 10.1038/srep24031 (PMC4832180; doi:10.1038/srep24031)
Supplement: Supplementary Information [file srep24031-s1.pdf]

Comparisons of parallel potential biomarkers of $_1$H-MRS-measured hepatic lipid content in patients with non-alcoholic fatty liver disease

Kai-Lun Shih[1†], Wei-Wen Su[1†], Chia-Chu Chang[2,3], Chew-Teng Kor[4] , Chen-Te Chou[5], Ting-Yu Chen[6], and Hung-Ming Wu[6,7,8*]

[1] Department of Gastroenterology, Changhua Christian Hospital, Changhua, Taiwan

[2] Department of Nephrology, Changhua Christian Hospital, Changhua, Taiwan

[3] School of Medicine, Chung-Shan Medical University, Taichung, Taiwan

[4] Internal Medicine Research Center, Changhua Christian Hospital, Changhua, Taiwan

[5] Department of Medical Imaging, Changhua Christian Hospital, Changhua, Taiwan

[6] Inflammation Research & Drug Development Center, Changhua Christian Hospital, Changhua, Taiwan

[7] Department of Neurology, Changhua Christian Hospital, Changhua, Taiwan

[8] Graduate Institute of Acupuncture Science, China Medical University, Taichung, Taiwan

[†] These two authors contributed equally to this work.

[*] ***Corresponding author and person to whom reprint requests should be addressed*:**

Hung-Ming Wu, MD, PHD

Inflammation Research & Drug Development Center

Changhua Christian Hospital

No. 135 Nan-Siau Street, Changhua 500, Taiwan

Tel: +886-4-7238595 ext. 4237

Fax: +886-4-7232942

E-mail: 18288@cch.org.tw

Supplementary Figure 1

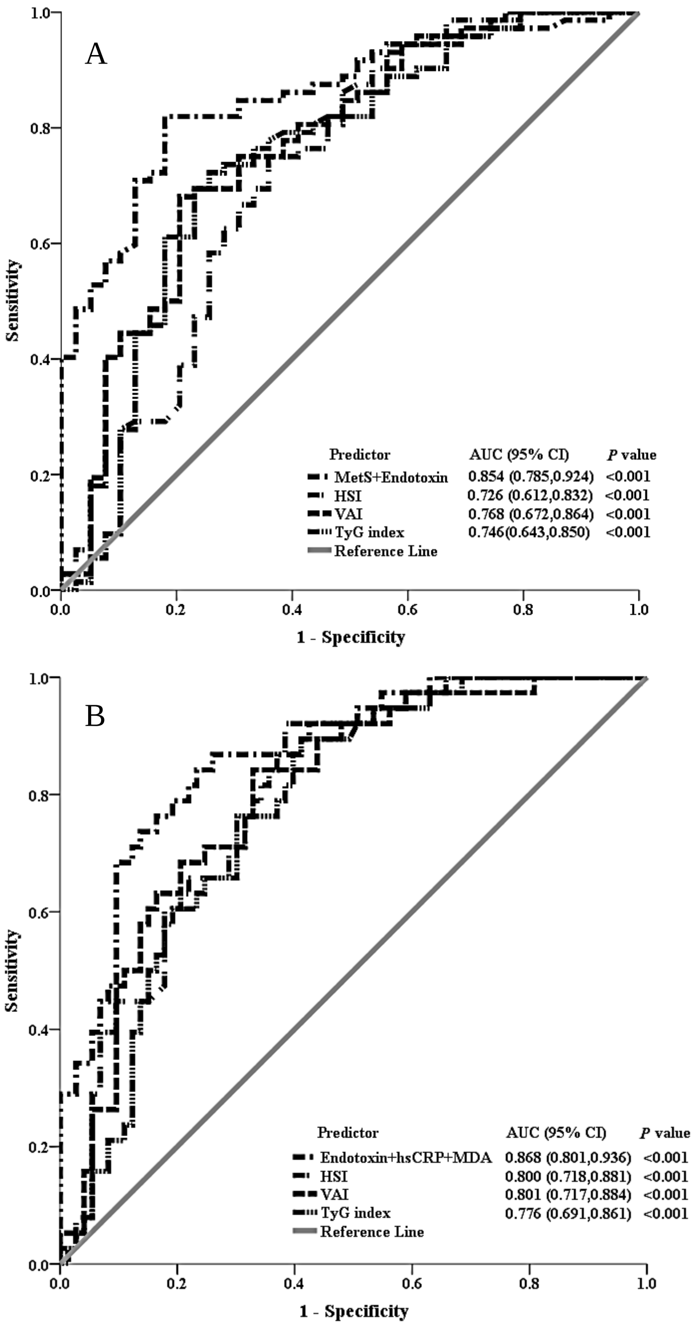

Supplementary Figure 1. Comparisons of ROC curves (AUCs) for HSI, VAI, TyG index, and the model in the present study. (A) AUCs of these four scoring systems for overall $_{1}$H-MRS-measured NAFLD, and (B) for $_{1}$H-MRS-measured NAFLD with liver injury. HSI, hepatic steatosis index includes ALT/AST, BMI, gender, and diabetes; TyG index, triglyceride x glucose index; VAI, visceral adiposity index includes waist circumference, triglyceride, HDL-C, and BMI.
